# Plasma Sphingolipid Profile Associated With Subclinical Atherosclerosis and Clinical Disease Markers of Systemic Lupus Erythematosus: Potential Predictive Value

**DOI:** 10.3389/fimmu.2021.694318

**Published:** 2021-07-21

**Authors:** Samar M. Hammad, Olivia C. Harden, Dulaney A. Wilson, Waleed O. Twal, Paul J. Nietert, Jim C. Oates

**Affiliations:** ^1^ Department of Regenerative Medicine & Cell Biology, Medical University of South Carolina, Charleston, SC, United States; ^2^ Medical University of South Carolina, Charleston, SC, United States; ^3^ Department of Public Health Sciences, Medical University of South Carolina, Charleston, SC, United States; ^4^ Department of Medicine, Division of Rheumatology & Immunology, Medical University of South Carolina, Charleston, SC, United States; ^5^ Medical Service, Rheumatology Section, Ralph H. Johnson VA Medical Center, Charleston, SC, United States

**Keywords:** sphingolipid, sphingomyelin, ceramide, sphingosine, lactosylceramide, lupus, LDL, atherosclerosis

## Abstract

Systemic lupus erythematosus (SLE) is a chronic autoimmune disease that affects females more than males, with African Americans developing more severe manifestation of the disease. SLE patients are at increased risk for cardiovascular disease (CVD), and SLE women 35-44 years old have 50 fold the incidence rate of CVD. Because SLE patients do not follow the typical age and gender pattern for CVD, but instead an accelerated disease course, the traditional biomarkers of elevated LDL and total cholesterol levels do not accurately assess their CVD risk. Recently, we have reported that African American SLE patients had higher ceramide, hexosylceramide, sphingosine and dihydrosphingosine 1-phosphate levels compared to their healthy controls, and those with atherosclerosis had higher sphingomyelin and sphingoid bases levels than those without (PLoS One. 2019; e0224496). In the current study, we sought to identify sphingolipid species that correlate with and pose the potential to predict atherosclerosis severity in African American SLE patients. Plasma samples from a group of African American predominantly female SLE patients with well-defined carotid atherosclerotic plaque burden were analyzed for sphingolipidomics using targeted mass spectroscopy. The data demonstrated that at baseline, plaque area and C3 values correlated inversely with most lactoceramide species. After one-year follow-up visit, values of the change of plaque area correlated positively with the lactoceramide species. There was no correlation between LDL-C concentrations and lactoceramide species. Taken together, lactocylcermide levels may have a ‘predictive’ value and sphingolipidomics have an added benefit to currently available tools in early diagnosis and prognosis of African American SLE patients with CVD.

## Introduction

Systemic lupus erythematosus (SLE) is a chronic autoimmune disease with various organ involvement and severity. The majority (90%) of lupus patients are females, and African American women are three times more likely than white women to have lupus and develop severe symptoms (1–4). Despite the dyslipidemia and accelerated cardiovascular disease (CVD) associated with SLE (5), the significance of the conventional plasma lipid panel (e.g., cholesterol and triglycerides) in the diagnosis/prognosis of CVD in SLE patients has been in question. For instance, the efficacy of statins to prevent atherosclerosis in SLE was found to be inconsistent (6). Furthermore, African Americans have increased risk of CVD although normally they have lower triglycerides and higher HDL cholesterol levels than other ethnicities (7).

Sphingolipids are both structural lipids and signaling molecules that are associated with cellular membranes and plasma lipoprotein, and their metabolism is tightly regulated to maintain homeostasis (8, 9). Sphingolipids in the blood are carried on circulating lipoprotein particles (HDL, LDL, and VLDL), and their use as disease biomarkers has been explored (9). Dysregulation of the sphingolipid pathway has been described in several inflammatory and immune-mediated diseases (10, 11), and alterations in the sphingolipid pathway in SLE and some of its related complications have been reported (12–16). A cross sectional study on a European SLE cohort showed that dysregulations in circulating sphingolipids is associated with clinical systemic disease activity and renal disease activity (17). In another cross-sectional study, lupus nephritis patients were stratified by severity of renal impairment, and the results showed that C16:0, C18:0, C20:0, and C24:1 ceramides were significantly elevated in the plasma of lupus nephritis patients when compared to healthy controls, and SLE patients without renal impairment (18). Ceramide C24:1 showed the most potential of being used as a biomarker of lupus nephritis, as it remained strongly elevated in lupus nephritis patients (*p* =0.0001), even when compared to SLE patients without kidney disease (18). These data show the potential value of assessing changes in circulating sphingolipids to identify a biomarker(s) for early detection of SLE and its comorbidities.

We have recently demonstrated that healthy African Americans have higher sphingomyelin (SM) levels and lower lactosylceramide (Lact-Cer) levels compared to healthy whites, and that SLE patients, irrespective of race, have higher levels of ceramide, and sphingoid bases [sphingosine and dihydrosphingosine (dhSph)] and their phosphates [sphingosine 1-phosphate (S1P) and dihydrosphingosine 1-phosphate (dhSph-1P)] compared to healthy participants (19). We also showed that compared to African American healthy controls, African American SLE patients have higher ceramide, hexosylceramide (Hex-Cer), sphingosine and dhSph-1P levels; and that African American SLE patients with atherosclerosis have higher sphingoid bases and SM levels compared to African American SLE patients without atherosclerosis (19). Notably, plasma levels of sphingosine, C16:0 ceramide/S1P ratio and C24:1 ceramide/S1P ratio significantly correlated with SLEDAI (Systemic Lupus Erythematosus Disease Activity Index) in the African American but not white SLE patients. In the present study, we investigated plasma sphingolipids as potential biomarkers that can predict or indicate atherosclerosis severity and/or established clinical SLE disease markers in African American SLE patients. Using targeted sphingolipidomics, plasma samples from a unique well-characterized African Americans lupus cohort with subclinical atherosclerosis (20, 21) were analyzed.

## Materials and Methods

### Study Participants

Banked plasma samples were utilized from participants who were previously recruited for a cross sectional within-lupus case-control study to evaluate novel and traditional risk factors for accelerated atherosclerosis in a largely African American SLE population (20). Fifty-one participants with SLE but without a history of clinical cardiovascular events were enrolled. At entry, participants met at least four of the 1997 revised American College of Rheumatology (ACR) SLE criteria (22). Traditional risk factors for atherosclerosis assessed by interview and chart review were history of major cardiovascular event or peripheral vascular disease (for exclusion criteria), number of years since SLE diagnosis, history of hypertension, diabetes, obesity, hypercholesterolemia, or smoking (current). Sera and plasma samples were analyzed for lipids and lupus activity markers. Urine protein, creatinine, and cell count; serum C3, C4, and anti-double-stranded DNA (dsDNA) antibody levels; complete blood count; complete metabolic panel; and plasma triglycerides, VLDL, LDL, and HDL cholesterol (VLDL-C, LDL-C, and HDL-C), and total cholesterol were analyzed at the CLIA-certified Medical University of South Carolina (MUSC) Clinical Chemistry, Hematology, and Immunology Laboratories. Medication histories were performed by interview and chart review; the following medications were recorded as either present or absent: immunomodulators (mycophenolate mofetil, mycophenolic acid, azathioprine, methotrexate, or hydroxychloroquine), angiotensin-converting enzyme (ACE) inhibitors, angiotensin receptor blockers (ARBs), prednisone and statins.

This cohort was then followed prospectively, and carotid ultrasound measurements were performed to determine total plaque area (TPA) in both carotids at baseline (visit 1) and one year later (visit 2). TPA was then reported as % TPA or a percent for age and sex-matched controls. Participants were labeled as having accelerated atherosclerosis (cases) if their age and sex-adjusted TPA was greater than the mean of historical controls in a hypertension stroke prevention clinic (23). The rationale for the use of carotid TPA as a marker of clinically relevant atherosclerosis and its measurement with ultrasound was explained previously (21). The historical control population in that study was used to reduce the confounding effect of age on TPA rather than to directly compare TPA in SLE and non-SLE populations (20). Because the goal in the initial study was to determine markers of subclinical atherosclerosis, participants with a history of clinical CVD such as myocardial infarction (MI); stroke (cerebrovascular accident, CVA); or documented peripheral, coronary, or carotid artery functionally significant narrowing were not included in the study. Those with a serum creatinine of > 3.0 were excluded due to the confounding effect of chronic kidney disease on the progression of atherosclerosis. Blood was collected at visits one and two (approximately 12 months apart), immediately processed for platelet-rich plasma, and frozen at -80°C for later, retrospective analysis. A subset (n =39) of the original (n =51) participants was selected for sphingolipid analysis, based upon the availability of their banked plasma samples.

### Sphingolipid Extraction and Analysis

Mass spectroscopy was used to measure plasma levels of individual species of five classes of sphingolipids: ceramides, sphingoid bases: sphingosine and dhSph and their phosphates (S1P and dhSph-1P, respectively), SM, and the glycosphingolipids Lact-Cer and Hex-Cer as previously described (24–29). Briefly, 100 μl of de-identified plasma sample (collected in EDTA as anti-coagulant and stored at -80°C) from each participant was spiked with internal standards and the sphingolipid complement in each sample was extracted. The sphingolipids in plasma extracts were separated and their masses quantitated using high performance liquid chromatography-tandem mass spectrometry (HPLC-MS/MS) at the MUSC Lipidomics Shared Resource. Lipids eluted during chromatography were detected and quantitated using a Thermo Scientific Quantum Access triple quadruple mass spectrometer equipped with an electrospray ion source (ESI) operating in multiple reaction monitoring (MRM) positive ion mode. Chromatographic separations were obtained under a gradient elution of a Peeke Scientific C8 Column (Redwood City, CA). Quantitative analyses were based on calibration curves generated by injecting known amounts of the target analytes and an equal amount of the internal standards. A listing of the internal standards used, and of the sphingolipids with available calibration standards was previously published (24). The calibration standards were obtained predominately from the MUSC Lipidomics Share Resource facility, and from commercially available sources, Avanti Polar Lipids Inc. and Matreya LLC. Molecular species of sphingolipids, which do not have available standards, were quantified using the calibration curve of the closest eluting counterpart. The final concentrations of analytes in the samples were determined using the appropriate corrections for sample loss based on internal standard recovery calculations. The resulting data was then normalized to the volume of sample analyzed. Final results were reported as pmol/ml plasma.

### Statistical Analyses

Descriptive statistics were used to characterize the study participants with respect to demographics and clinical variables. Because of non-normality of many of the study variables, Spearman rank correlations were used to assess associations between sphingolipids and measures of atherosclerosis, and correlations with *p*-values <0.05 were highlighted. For correlations assessing change in total plaque area, partial Spearman correlations that adjusted for baseline total plaque area were also reported. With a sample size of n =39 participants, our study had 80% power to detect correlations as small as 0.44 with 2-sided testing and alpha=0.05. Wilcoxon rank sum tests were used to compare baseline sphingolipid levels between patients where were (n =4) and were not (n =35) on statins throughout the course of the follow-up time period. *p-values* < 0.05 were considered statistically significant, and as this was largely a hypothesis generating study, no adjustments were made for multiple hypothesis testing. Analyses were conducted using SAS v9.4 (SAS Institute, Cary, NC).

## Results

### Baseline Clinical Characteristics and Plasma Sphingolipidomics

All of the SLE patients of this study (n =39) were African American, and most were (90%) females. The clinical characteristics at baseline (visit 1) are summarized in **Table 1**. The mean age at baseline was 43 years, with an average of 10-year duration of SLE. Serum concentrations of triglycerides and cholesterol in lipoprotein fractions were within the normal range **(** **Table 1** **)** and were similar to those formerly reported for their matched controls (21). Notably, the mean total cholesterol was found to be 10% lower among SLE patients compared with the matched controls (21); hydroxychloroquine, the drug that may have been used to treat the SLE patients before baseline, was previously shown to lower total cholesterol levels by about 15% after starting treatment (30).

**Table 1:** Baseline (visit 1) demographics and clinical characteristics.

Variable	Mean	Standard Deviation	Median	IQR	
				From	To
**Age (yrs.)**	43.1	± 13.0	45.8	28.1	55.3
**Years with SLE (yrs.)**	10.1	± 8.1	7.0	4.3	14.5
Total plaque area (% of controls)* at baseline	80.4	± 114.3	54.5	0.0	97.0
**Total plaque area (cm^2^) at visit 1 (baseline)**	0.29	± 0.41	0.12	0.00	0.45
**Total plaque area (cm^2^) at visit 2**	0.17	± 0.34	0.00	0.00	0.19
Change in plaque (cm^2^)**	-0.12	± 0.44	0.00	-0.15	0.00
**Blood pressure: systolic**	131.7	± 21.1	130.0	116.0	144.0
**Blood pressure: diastolic**	78.2	± 13.9	77.0	66.0	89.0
**Waist-hip ratio**	0.8	± 0.2	0.8	0.8	0.9
**SLEDAI score**	4.1	± 3.0	4.0	2.0	6.0
**SLICC score**	1.2	± 1.5	1.0	0.0	2.0
**LDL-C (mg/dl)**	106.8	± 39.4	102.0	77.0	124.0
**HDL-C (mg/dl)**	47.1	± 12.7	46.0	38.0	55.0
**Triglycerides (mg/dl)**	93.7	± 39.8	91.0	60.0	118.0
**C3 (mg/dl)**	104.2	± 27.7	106.5	89.6	125.2
**C4 (mg/dl)**	18.0	± 5.7	17.4	13.6	22.8
**dsDNA antibodies (IU/ml)**	140.9	± 184.3	76.7	4.9	221.3
**Urine protein/creatinine**	0.3	± 0.4	0.1	0.1	0.2

N = 39 (Females: 35 (89.7%), Males: 4 (10.3%), All African Americans.

*% of age- and sex-matched historical controls.

**Absolute change in plaque from baseline (visit 1) to visit 2 (approximately 12 months apart).

IQR, Interquartile range; SLEDAI, Systemic lupus erythematosus disease activity index; SLICC, Systemic Lupus International Collaborating Clinics; LDL-, HDL-C; HDL-, LDL-cholesterol.

The descriptive statistics of the baseline plasma sphingolipids for the large subset of SLE patients (n =35) who were not consistently on statins throughout the study are presented in **Table 2**. The mean ± SD and the median with IQR of the measured medium-, long- and very long-chain SM, ceramide, and glycosphingolipid (Lact-Cer and Hex-Cer) species, and sphingoid bases (sphingosine and dhSph) and their phosphates (S1P and dhSph-1P, respectively) are reported, as are the C16:0 ceramide/S1P, C24:1 ceramide/S1P, C16:0 ceramide/C24:0 ceramide, C18:0 ceramide/C24:0 ceramide, and C24:1 ceramide/C24:0 ceramide ratios **(** **Table 2** **)**. The baseline plasma sphingolipids of all participating SLE patients (n =39) and those who used statins consistently over the period extending from visit 1 to visit 2 (n =4) are presented in **Table 4**. The mean sphingolipid levels did not differ by hydroxylchloroquine use or by prednisone dose (data not shown). However, for those patients who used ACE inhibitors (N =9), levels of C14:0, C16:0, C18:0, C22:1, C24:1 ceramides and C16:0 ceramide/S1P ratio were significantly higher (*p*<0.05) than in those who did not (**Table S1**). For those who used ARBs (N =3), C16:0 ceramide/C24:0 ceramide, C18:0 ceramide/C24:0 ceramide, and C24:1 ceramide/C24:0 ceramide ratios were significantly higher (*p*<0.05), but levels of C20:0 and C24:0 SM were lower (*p*<0.05) than in those who did not (**Table S2**).

**Table 2:** Baseline descriptive statistics of plasma sphingolipids among study participants (n =35) not consistently on statins throughout the study.

Sphingolipids [pmol/100 µl plasma]	Mean	Standard Deviation	Median	IQR	
				From	To
**Sphingomyelin (SM)**					
C14:0 SM	1041.00	± 388.85	916.53	808.4	**1196.4**
C16:0 SM	18500.00	± 3788.00	17611.00	**15786.1**	21503.6
C18:0 SM	1340.00	± 218.17	1314.00	1157.1	1489.1
C18:1 SM	665.61	± 132.24	655.66	554.3	735.5
C20:0 SM	720.25	± 118.82	695.23	642.7	809.7
C20:1 SM	294.20	± 48.57	291.66	257.5	327.4
C22:0 SM	1417.00	± 239.45	1413.00	1260.7	1502.7
C22:1 SM	1082.00	± 147.11	1080.00	956.2	1169.4
C24:0 SM	1217.00	± 237.37	1202.00	1033.3	1355.6
C24:1 SM	2966.00	± 344.44	3037.00	2697.7	3316.0
C26:0 SM	8.57	± 1.96	8.60	7.1	9.9
C26:1 SM	21.12	± 5.25	20.61	16.7	24.7
Total SM	29272.00	± 4667.00	28279.00	25652.7	33846.5
**Ceramide (Cer)**					
C14:0 Cer	3.24	± 0.96	3.00	2.7	3.6
C16:0 Cer	53.35	± 20.74	52.02	40.2	59.9
C18:0 Cer	16.42	± 7.12	14.64	12.7	20.9
C18:1 Cer	5.13	± 2.67	4.40	3.2	6.3
C20:0 Cer	33.69	± 14.90	31.52	26.4	38.4
C20:1 Cer	6.22	± 2.49	5.94	4.4	7.7
C20:4 Cer	0.04	± 0.03	0.04	0.0	0.1
C22:0 Cer	138.76	± 34.06	140.04	110.5	153.6
C22:1 Cer	50.73	14.23	47.73	41.4	63.7
C24:0 Cer	582.25	± 197.05	555.36	477.3	703.7
C24:1 Cer	228.19	± 64.18	236.11	193.8	276.0
C26:0 Cer	18.36	± 9.94	17.13	10.8	22.9
C26:1 Cer	9.87	± 4.44	9.66	7.2	12.5
Total Cer	1146.00	± 327.38	1147.00	973.2	1393.8
**Dihydro-C16:0 Cer**	2.12	± 1.05	1.83	1.4	2.7
**Lactosylceramide (Lact-Cer)**					
C14:0 Lact-Cer	9.64	± 4.29	10.23	6.6	11.4
C16:0 Lact-Cer	265.62	± 91.51	272.41	186.7	319.0
C18:0 Lact-Cer	11.41	± 4.53	10.55	8.2	13.2
C18:1 Lact-Cer	8.07	± 4.45	6.23	4.4	11.2
C20:0 Lact-Cer	2.84	± 1.19	2.72	2.1	3.2
C20:1 Lact-Cer	0.29	± 0.17	0.24	0.2	0.4
C22:0 Lact-Cer	8.78	± 3.55	8.34	6.1	10.5
C22:1 Lact-Cer	0.57	± 0.25	0.50	0.4	0.7
C24:0 Lact-Cer	2.50	± 0.88	2.29	1.8	3.0
C24:1 Lact-Cer	32.01	± 11.91	30.34	24.4	37.6
C26:0 Lact-Cer	0.11	± 0.06	0.11	0.1	0.1
C26:1 Lact-Cer	0.11	± 0.04	0.11	0.1	0.1
Total Lact-Cer	341.95	± 113.27	323.16	247.7	420.7
**Hexosylceramide (Hex-Cer)**					
C14:0 Hex-Cer	0.57	± 0.27	0.50	0.3	0.7
C16:0 Hex-Cer	76.81	± 28.36	75.70	57.1	95.4
C18:0 Hex-Cer	0.56	± 0.25	0.49	0.4	0.6
C18:1 Hex-Cer	0.19	± 0.08	0.19	0.1	0.2
C20:0 Hex-Cer	0.96	± 0.38	0.87	0.7	1.1
C20:1 Hex-Cer	0.12	± 0.07	0.11	0.1	0.1
C22:0 Hex -Cer	41.51	± 11.16	42.26	34.9	49.0
C22:1 Hex -Cer	1.51	± 0.52	1.46	1.0	1.8
C24:0 Hex -Cer	57.27	± 15.32	56.69	44.9	74.0
C24:1 Hex -Cer	72.97	± 23.39	68.81	55.5	88.7
C26:0 Hex -Cer	0.90	± 0.37	0.88	0.6	1.2
C26:1 Hex -Cer	0.46	± 0.19	0.40	0.3	0.6
Total Hex-Cer	253.83	± 66.38	234.1	205.53	296.64
**Dihydrosphingosine (dhSph)**	0.58	± 0.26	0.53	0.4	0.7
**Sphingosine**	1.86	± 0.76	1.58	1.3	2.3
**dhSph 1-phosphate (dhSph-1P)**	16.18	± 3.95	16.48	13.3	19.5
**Sphingosine 1-phosphate (S1P)**	60.17	± 12.55	56.99	51.0	65.5
**C16:0 Cer: S1P Ratio**	0.92	± 0.39	0.91	0.6	1.1
**C24:1 Cer: S1P Ratio**	3.91	± 1.28	3.97	3.0	4.5
**C16:0 Cer: C24:0 Cer Ratio**	0.095	± 0.03	0.09	0.07	0.12
**C18:0 Cer: C24:0 Cer Ratio**	0.03	± 0.1	0.03	0.02	0.04
**C24:1 Cer: C24:0 Cer Ratio**	0.41	± 0.07	0.37	0.34	0.46

N = 35, not consistently on statins throughout the study.

IQR, Interquartile range.

At visit 1, the mean TPA for this study cohort was about 80% of the mean TPA of age- and sex-matched historical controls. One year after visit 1 (i.e., visit 2), TPA (cm^2^) was minimally reduced but not with statistical significance [mean ± SD: -0.12 ±0.44; median with interquartile range (IQR): 0.00 (-0.15, 0.00)]; 51.3% of the patients experienced declines in TPA during the course of the study. As noted in **Table 1**, there is great heterogeneity in the lupus population, with some patients have lower levels than controls and some have higher levels. In addition, the control participants were “disease” controls with hypertension in a prevention clinic. As shown also in **Table 5**, the SLEDAI scores in this cohort correlate negatively with Systemic Lupus International Collaborating Clinics Damage Index (SLICC) scores, as well as C3 and C4 levels, whereas SLICC scores correlate positively with C3 and C4 levels. Levels of dsDNA antibodies were found to correlate negatively with SLICC scores, and also with C3 and C4 levels. Characteristically, early SLE markers (e.g., dsDNA) are associated with disease activity, whereas later SLE markers (e.g., low C3 and C4 levels) are associated with organ damage (31). Notably, as shown in **Table 5**, levels of plasma triglycerides, LDL-C, and HDL-C were found not to correlate with any of the traditional SLE makers (SLEDAI, SLICC, C3, C4, dsDNA).

### Lactosylceramides Correlate Negatively With Plaque Area and C3, While Sphingomyelins, Ceramides, and Hexosylceramides Correlate Positively With LDL-C

Correlations between concentrations of plasma sphingolipids and clinical variables, including baseline (visit 1) plaque and change in plaque at visit 2 are presented in **Table 3**. For this correlation analysis, participants on statins at both visits (n =4) were excluded, since the statins themselves may impact sphingolipids (32). At baseline, TPA values are shown to correlate negatively with the concentrations of the two most dominant Lact-Cer species (C16:0 and C24:1), Lact-Cer species with long-chain saturated fatty acids (C20:0, C22:0, and C24:0), and total Lact-Cer. However, TPA values are shown to correlate positively with the concentrations of C20:0, C22:0, and total SM, and the very long-chain ceramide species C24:0, C26:0 and C26:1 **(** **Table 3** **)**. At visit 2, change in TPA from baseline is shown to correlate positively with the two dominant Lact-Cer species (C16, and C24:1) and total Lact-Cer, but negatively with C14:0 SM concentration. No statistically significant correlations were found between the values of the change of TPA from baseline and concentrations of ceramide and Hex-Cer species; however, negative correlations were found with concentrations of sphingosine, dhSph and dhSph-1P. After adjusting for age (using partial correlation), the correlation between change in plaque area and the sphingolipids became non-significant for C14:0 SM, C20:0 SM, C14:0 Lact-Cer, and C16:0 Hex-Cer.

**Table 3:** Correlations between plasma sphingolipids and clinical variables, including baseline (visit 1) plaque area and change in plaque at visit 2.

Sphingolipids	Total plaque area %*	Change in Plaque**	Age	Years with SLE	BP Syst.	BP Diast.	Waist-hip ratio		SLEDAI score	SLICC score	LDL-C	HDL-C	TG	C3	C4	dsDNA	Urine protein/creatinine
**Sphingomyelin (SM)**																	
C14:0 SM	0.30	***-0.35***	0.26	-0.02	0.03	0.03	0.13	0.15		-0.09	***0.77***	0.33	0.32	0.10	-0.11	-0.002	0.30
C16:0 SM	0.28	-0.02	0.08	0.01	0.01	0.19	0.15	-0.06		0.08	***0.83***	0.24	***0.39***	-0.05	0.08	0.01	***0.35***
C18:0 SM	0.03	-0.12	0.02	-0.16	-0.24	-0.07	-0.02	-0.09		-0.003	***0.60***	0.10	0.31	0.05	0.16	-0.02	-0.14
C18:1 SM	0.04	-0.04	0.25	0.02	-0.05	-0.10	-0.05	-0.23		0.30	***0.52***	0.11	***0.44***	0.26	0.26	-0.11	0.04
C20:0 SM	***0.41***	-0.28	***0.47***	***0.35***	-0.06	-0.12	0.13	0.09		-0.02	***0.43***	0.28	0.09	***0.36***	0.05	-0.08	-0.12
C20:1 SM	0.23	-0.16	***0.47***	0.25	0.02	-0.20	-0.19	-0.09		0.31	***0.35***	0.23	0.24	***0.37***	0.12	-0.11	-0.03
C22:0 SM	***0.35***	-0.00	***0.34***	0.30	-0.005	0.11	0.32	0.07		0.01	***0.57***	0.15	0.22	0.26	0.07	-0.09	-0.05
C22:1 SM	0.15	-0.25	***0.51***	0.27	0.10	-0.08	0.21	-0.08		0.24	***0.43***	0.10	0.32	***0.45***	0.10	-0.08	0.10
C24:0 SM	0.26	0.23	***0.35***	0.29	0.19	0.22	0.18	-0.01		0.20	0.30	0.20	0.01	0.23	0.12	-0.19	-0.18
C24:1 SM	0.27	0.24	***0.36***	0.30	0.01	-0.17	0.09	-0.25		0.31	***0.36***	0.15	0.13	0.22	0.23	-0.30	0.04
C26:0 SM	-0.005	0.15	-0.02	0.11	0.14	***0.38***	-0.07	0.11		0.09	0.01	0.17	-0.10	-0.20	-0.10	0.05	-0.19
C26:1 SM	0.24	0.22	0.21	0.17	0.03	0.10	0.05	-0.17		0.18	0.28	0.14	0.04	-0.07	0.15	-0.15	-0.09
Total SM	***0.34***	-0.09	0.20	0.09	0.002	0.13	0.19	-0.08		0.13	***0.88***	0.23	***0.44***	0.10	0.16	-0.07	0.29
**Ceramide (Cer)**																	
C14:0 Cer	0.06	-0.06	0.13	0.02	-0.03	0.25	0.26	-0.13		0.12	***0.69***	-0.05	0.31	0.08	0.02	0.09	0.31
C16:0 Cer	-0.01	0.25	-0.04	-0.11	0.04	0.31	0.27	-0.15		0.22	***0.55***	-0.09	0.27	-0.14	0.08	0.27	***0.47***
C18:0 Cer	0.13	0.05	0.04	0.03	0.01	0.13	0.14	-0.11		0.16	***0.58***	0.04	***0.41***	0.10	0.09	0.18	0.23
C18:1 Cer	0.15	-0.04	0.20	0.03	0.10	0.09	0.10	-0.18		0.30	***0.64***	0.11	***0.40***	0.24	0.21	0.07	0.19
C20:0 Cer	0.15	-0.10	***0.36***	0.30	0.23	***0.35***	0.15	0.05		0.25	***0.51***	0.22	***0.44***	0.33	0.09	0.06	0.32
C20:1 Cer	0.18	0.00	***0.39***	0.20	0.12	0.10	0.07	-0.14		***0.37***	***0.57***	0.22	***0.40***	0.33	0.24	0.05	0.24
C20:4 Cer	0.26	-0.03	-0.005	-0.06	-0.06	-0.02	0.03	-0.12		0.15	0.18	0.25	-0.12	0.23	0.11	-0.14	0.22
C22:0 Cer	0.001	0.07	-0.06	0.02	0.02	0.27	0.30	0.02		0.11	***0.65***	0.14	***0.36***	0.12	0.17	0.02	0.09
C22:1 Cer	0.13	-0.14	0.18	0.08	0.01	0.18	0.28	-0.09		0.15	***0.72***	0.04	***0.51***	0.28	0.17	0.05	0.26
C24:0 Cer	***0.36***	-0.01	0.18	0.11	0.05	0.20	0.32	-0.07		0.04	***0.70***	0.19	0.26	0.19	0.22	-0.03	0.08
C24:1 Cer	0.13	0.02	0.07	0.10	-0.09	0.03	0.28	-0.23		0.16	***0.57***	-0.02	0.33	0.25	0.26	-0.05	0.21
C26:0 Cer	***0.42***	-0.01	0.11	-0.03	0.03	0.20	0.29	-0.14		0.02	***0.57***	0.14	0.18	0.06	0.32	-0.02	0.02
C26:1 Cer	***0.46***	0.13	0.15	0.03	0.04	0.15	0.18	-0.26		0.16	***0.47***	0.08	0.20	0.03	***0.36***	-0.03	0.17
Total Cer	0.29	0.01	0.15	0.11	0.03	0.19	0.33	-0.08		0.10	***0.72***	0.14	***0.35***	0.20	0.22	0.04	0.21
**Dihydro-C16:0 Cer**	0.16	-0.00	-0.10	0.02	-0.07	0.26	0.15	0.09		0.04	***0.76***	0.25	0.19	-0.13	0.11	0.10	0.25
**Lactosylceramide (Lact-Cer)**																	
C14:0 Lact-Cer	-0.26	0.27	-0.21	-0.16	-0.14	-0.05	-0.17	0.03		-0.02	0.23	0.20	-0.07	***-0.35***	-0.28	0.20	0.02
C16:0 Lact-Cer	***-0.35***	***0.45***	***-0.38***	-0.20	-0.10	0.11	-0.05	-0.02		-0.01	0.18	0.05	-0.19	-0.33	-0.14	0.15	-0.14
C18:0 Lact-Cer	-0.18	0.12	-0.23	-0.25	-0.01	0.11	-0.12	-0.06		0.10	0.15	-0.04	-0.12	***-0.44-0.44***	-0.18	0.12	0.04
C18:1 Lact-Cer	-0.07	0.25	-0.04	0.04	0.13	0.23	-0.02	-0.03		***0.34***	0.32	0.03	0.12	-0.19	0.07	0.01	0.17
C20:0 Lact-Cer	***-0.38***	0.04	***-0.36***	-0.10	-0.24	0.01	0.06	0.22		-0.04	-0.09	-0.24	-0.16	***-0.39***	-0.26	0.30	0.01
C20:1 Lact-Cer	-0.22	0.16	-0.11	-0.17	0.21	0.24	-0.10	0.11		-0.06	-0.15	-0.20	-0.02	***-0.47***	-0.31	0.25	0.17
C22:0 Lact-Cer	***-0.43***	0.23	***-0.36***	-0.21	-0.12	-0.02	0.12	0.19		-0.15	-0.05	-0.16	-0.20	***-0.35***	-0.30	0.25	0.13
C22:1 Lact-Cer	-0.31	0.22	***-0.42***	-0.19	-0.09	0.01	-0.04	0.21		-0.01	0.02	0.05	-0.28	***-0.46***	***-0.34***	0.20	0.13
C24:0 Lact-Cer	***-0.40***	0.15	***-0.39***	-0.27	-0.22	-0.19	0.09	0.24		***-0.34***	-0.18	-0.13	-0.26	***-0.44***	***-0.48***	0.24	0.06
C24:1 Lact-Cer	***-0.36***	***0.41***	***-0.46***	-0.22	-0.25	-0.14	0.09	0.01		-0.06	-0.08	0.01	***-0.40***	***-0.32***	-0.21	0.04	-0.14
C26:0 Lact-Cer	-0.14	0.25	-0.03	-0.16	0.004	0.21	-0.09	0.07		-0.25	***0.40***	0.13	0.01	***-0.34***	-0.32	0.27	0.16
C26:1 Lact-Cer	0.01	0.31	-0.09	-0.06	-0.20	-0.04	-0.01	-0.06		-0.08	0.10	0.04	0.03	-0.05	-0.003	-0.01	0.09
Total Lact-Cer	***-0.35***	***0.45***	***-0.36***	-0.23	-0.14	0.05	-0.04	-0.01		-0.02	0.18	0.05	-0.19	-0.33	-0.19	0.15	-0.11
**Hexosylceramide (Hex-Cer)**																	
C14:0 Hex-Cer	0.03	-0.11	-0.26	-0.07	-0.29	-0.05	-0.12	0.06		-0.06	***0.50***	0.28	0.24	***-0.34***	-0.11	0.07	0.15
C16:0 Hex-Cer	-0.28	0.20	***-0.40***	-0.23	-0.09	0.26	0.01	0.07		0.05	***0.49***	0.17	0.06	-0.32	-0.04	0.21	0.15
C18:0 Hex-Cer	0.16	-0.08	-0.14	0.04	***-0.43***	***-0.34***	-0.21	0.03		***-0.36***	0.25	0.11	-0.20	-0.26	***-0.34***	-0.06	***-0.34***
C18:1 Hex-Cer	-0.04	0.02	-0.15	0.11	-0.25	-0.15	-0.14	0.13		-0.05	***0.43***	0.19	0.15	-0.24	-0.26	0.19	-0.04
C20:0 Hex-Cer	0.001	0.03	***-0.40***	-0.05	***-0.40***	-0.17	-0.13	0.14		***-0.38***	0.20	0.27	-0.30	***-0.35***	-0.21	0.09	-0.23
C20:1 Hex-Cer	-0.08	-0.05	-0.31	-0.05	-0.24	-0.15	-0.24	0.08		-0.13	0.25	0.01	0.04	***-0.43***	-0.09	0.003	-0.16
C22:0 Hex -Cer	-0.02	0.13	-0.31	-0.20	-0.19	0.15	0.08	0.33		-0.29	***0.45***	0.21	-0.04	-0.28	-0.21	0.07	-0.06
C22:1 Hex -Cer	0.27	-0.16	-0.14	0.12	-0.21	-0.16	-0.05	0.08		-0.19	***0.46***	0.16	0.16	-0.14	-0.18	-0.12	-0.01
C24:0 Hex -Cer	0.02	0.13	-0.18	-0.14	-0.31	-0.02	0.11	0.18		***-0.33***	***0.45***	0.17	0.05	-0.28	-0.18	0.06	-0.003
C24:1 Hex -Cer	0.002	0.07	-0.31	-0.11	-0.29	-0.16	0.002	0.07		-0.24	***0.39***	0.16	-0.07	-0.20	-0.18	-0.09	-0.07
C26:0 Hex -Cer	0.20	-0.02	0.03	-0.05	-0.19	0.05	0.27	0.10		-0.26	***0.79***	0.22	0.27	-0.11	-0.03	-0.01	0.03
C26:1 Hex -Cer	0.27	-0.08	-0.09	0.13	-0.18	-0.09	0.01	0.01		-0.13	***0.50***	0.11	0.14	-0.10	-0.01	-0.14	0.09
Total Hex-Cer	-0.10	0.10	***-0.35***	-0.21	-0.23	0.04	-0.01	0.15		-0.20	***0.53***	0.25	0.01	-0.33	-0.18	0.05	0.02
**Dihydrosphingosine (dhSph)**	-0.01	***-0.42***	0.02	0.02	-0.06	0.05	0.03	0.27		-0.12	***0.59***	-0.12	***0.48***	-0.10	-0.21	0.14	0.17
**Sphingosine**	-0.10	***-0.40***	0.13	0.12	0.06	0.04	0.004	0.09		0.09	***0.39***	-0.24	***0.52***	0.15	-0.08	0.004	-0.001
**dhSph 1-phosphate (dhSph-1P)**	-0.16	***-0.41***	-0.21	-0.05	-0.31	-0.07	-0.10	0.18		-0.32	0.20	-0.22	0.29	-0.25	-0.30	0.05	-0.15
**Sphingosine 1-phosphate (S1P)**	-0.04	-0.22	0.03	0.17	-0.07	0.02	0.20	-0.03		0.005	0.10	-0.25	0.31	0.21	0.04	-0.07	-0.24
**C16:0 Cer: S1P Ratio**	-0.06	0.30	-0.12	-0.20	0.15	***0.36***	0.15	-0.03		0.20	***0.38***	0.04	0.12	-0.29	0.03	0.30	***0.52***
**C24:1 Cer: S1P Ratio**	0.04	0.18	0.03	0 .005	-0.005	0.01	0.15	-0.19		0.21	***0.40***	0.08	0.11	0.03	0.21	0.04	***0.37***
**C16:0 Cer: C24:0 Cer Ratio**	***-0.44***	***0.45***	-0.27	-0.24	0.01	0.19	-0.11	0.00		0.19	-0.09	-0.18	0.00	-0.29	-0.21	0.23	0.25
**C18:0 Cer: C24:0 Cer Ratio**	-0.18	0.16	-0.04	0.03	0.01	0.05	-0.23	-0.12		0.28	0.04	-0.11	0.23	-0.01	-0.08	0.07	0.20
**C24:1 Cer: C24:0 Cer Ratio**	-0.33	0.18	-0.14	0.01	-0.01	-0.11	-0.17	-0.24		0.22	-0.30	-0.31	0.13	0.06	0.05	-0.16	0.15

N =35. Data presented are correlations, where **bold italics** is statistically significant at < 0.05 **(black)**, < 0.01 **(red)**, < 0.001 **(blue)**, < 0.00001 **(violet)**, & Underlined: p=0.051 to p=0.059.

*% of age- and sex-matched historical controls. **Absolute change in plaque from baseline (visit 1) to visit 2. BP, blood pressure; Syst., systolic; Diast., diastolic; SLEDAI, Systemic lupus erythematosus disease activity index; SLICC, Systemic Lupus International Collaborating Clinics; LDL-; HDL-C, HDL-; LDL-cholesterol; TG, triglycerides.

The data in **Table 3** show that, in this study cohort, LDL-C concentrations strongly and positively correlate with concentrations of the majority of SM, ceramide, and Hex-Cer species, as well as C16:0 dihydroceramide, sphingosine, dhSph, C16:0 Cer/S1P and C24:1 Cer/S1P. In contrast, there is no significant correlations identified between concentrations of LDL-C and those of Lact-Cer species, except for C26:0 Lact-Cer, which exists in the circulation in a barely detectable amount **(** **Table 1** **)**. As shown in **Table 3**, there is no significant correlation present between HDL-C concentration and the concentration of any of the plasma sphingolipids.

**Table 3** also show that triglyceride concentrations correlate positively with concentrations of SM (C16:0, C18:1, and total), ceramide (C18:0, C18:1, C20:0, C20:1, C22:0, C22:1, and total), dh-Sph and sphingosine. However, triglyceride concentrations were found to have significantly negative correlation with the second dominant Lact-Cer species (C24:1 Lact-Cer), with statistically non-significant negative correlations with the concentrations of the remaining of Lact-Cer species. As shown in **Table 3**, triglyceride concentrations do not correlate with of any of the Hex-Cer species concentrations.

In this study, the older the patient the higher the concentrations of SM species (C20:0, C20:1, C22:0, C22:1, C24:0, and C24:1) and ceramide species (C20:0 and C20:1), and the lower the concentrations of Lact-Cer species (C16:0, C20:0, C22:0, C22:1, C24:0, C24:1, and total Lact-cer) and Hex-Cer species (C16:0, C20:0, and total Hex-Cer) **(** **Table 3** **)**. The duration of SLE prior to visit 1, concentrations of dsDNA antibodies, and SLEDAI scores, do not seem to have a significant effect on the concentrations of plasma sphingolipids. However, C3 concentrations were found to correlate negatively with almost all Lact-Cer species, and positively with three long-chain SM species. C4 concentrations, on the other hand, showed associations with concentrations of only few scarce plasma sphingolipid species **(** **Table 3** **)**.

### Circulating C24:1 Ceramide in African American with SLE Could Be Elevated in Response to Statins

Although this study was not purposely designed to study the effect of statins on the development of atherosclerosis in African American SLE patients, we compared the baseline (visit 1) concentrations of plasma sphingolipids in patients who consistently used statins over the period extending from visit 1 to visit 2 (n =4) with those were not indicated as being prescribed any statins (n =35). The data in **Table 4** show that C24:1 ceramide and C24:1 ceramide/S1P ratio were significantly higher in the former group of patients. The concentration of C26:0 Lact-Cer, one of the least detectable plasma sphingolipid species, was also higher in the SLE patients who consistently used statins compared to those who did not **(** **Table 4** **)**.

**Table 4:** Comparisons in plasma sphingolipids between patients who did versus did not use statins consistently from visit 1 to visit 2.

Sphingolipids [pmol/100 µl plasma]	Statins use not noted or not consistent N = 35	Statins use noted at baseline & visit 2 N = 4*	All participants at baseline N = 39	*P value*
**Sphingomyelin (SM)**				
C14:0 SM	916.53 (808.35, 1196.43)	1275.78 (1032.84, 1385.57)	931.08 (818.61, 1224.12)	0.16
C16:0 SM	17610.51 (15786.12, 21503.61)	20776.01 (16950.38, 24063.89)	17610.51 (16006.07, 22298.84)	0.29
C18:0 SM	1313.67 (1157.13, 1489.11)	1305.51 (1202.01, 1515.85)	1313.67 (1176.7, 1489.11)	0.85
C18:1 SM	655.66 (554.3, 735.49)	658.74 (551.3, 788.96)	655.66 (554.3, 745.75)	0.82
C20:0 SM	695.23 (642.72, 809.72)	722.95 (663.28, 753.61)	696.55 (642.72, 776.23)	0.96
C20:1 SM	291.66 (257.5, 327.39)	288.9 (256.95, 337)	291.66 (257.5, 327.39)	0.93
C22:0 SM	1413.17 (1260.69, 1502.66)	1305.04 (1053.79, 1565.89)	1413.17 (1250.71, 1503.8)	0.71
C22:1 SM	1079.84 (956.19, 1169.35)	1079.04 (1002.3, 1115.32)	1079.84 (956.19, 1161.2)	0.82
C24:0 SM	1202.42 (1033.28, 1355.55)	1160.99 (970.69, 1503.12)	1202.42 (1028.03, 1355.55)	0.78
C24:1 SM	3037.07 (2697.7, 3316.04)	3053.71 (2847.12, 3389.29)	3037.07 (2729.04, 3316.04)	0.43
C26 SM	8.6 (7.11, 9.93)	7.75 (7.08, 9.1)	8.3 (7.11, 9.93)	0.68
C26:1 SM	20.61 (16.69, 24.72)	21.32 (19.16, 23.84)	20.61 (16.95, 24.72)	0.71
Total SM	28279.45 (25652.71, 33846.53)	31324.86 (27721.74, 35286.62)	28279.45 (25889.17, 34238.7)	0.29
**Ceramide (Cer)**				
C14:0 Cer	3 (2.69, 3.56)	3.68 (2.97, 4.19)	3.08 (2.69, 3.56)	0.31
C16:0 Cer	52.02 (40.17, 59.92)	68.08 (47.76, 89.24)	52.5 (40.17, 61.65)	0.27
C18:0 Cer	14.64 (12.73, 20.86)	19.37 (11.3, 25.38)	14.72 (12.73, 21.36)	0.52
C18:1 Cer	4.4 (3.22, 6.27)	6.6 (3.59, 8.65)	4.62 (3.22, 6.55)	0.43
C20:0 Cer	31.52 (26.38, 38.41)	40.54 (29.37, 45.03)	31.55 (26.38, 40.21)	0.33
C20:1 Cer	5.94 (4.42, 7.69)	8.2 (4.86, 10.21)	6.11 (4.42, 8.22)	0.31
C20:4 Cer	0.04 (0.02, 0.05)	0.03 (0.01, 0.03)	0.04 (0.02, 0.05)	0.23
C22:0 Cer	140.04 (110.52, 153.59)	147.93 (117.7, 185.07)	140.04 (110.52, 154.65)	0.55
C22:1 Cer	47.73 (41.4, 63.73)	54.95 (49.94, 66.44)	48.85 (42.47, 63.73)	0.25
C24:0 Cer	555.36 (477.31, 703.67)	718.61 (557.31, 843.1)	557.2 (500.52, 722.32)	0.25
C24:1 Cer	236.11 (193.81, 276.02)	289.37 (265.97, 340.6)	241.5 (194.18, 285.11)	***0.033***
C26:0 Cer	17.13 (10.75, 22.87)	23.06 (20.99, 23.12)	17.51 (12.53, 23.11)	0.13
C26:1 Cer	9.66 (7.16, 12.53)	11.67 (10.39, 12.5)	9.69 (7.41, 12.53)	0.25
Total Cer	1147.36 (973.23, 1393.75)	1351.33 (1186.5, 1589.23)	1166.66 (979.87, 1393.84)	0.13
**Dihydro-C16:0 Cer**	1.83 (1.36, 2.73)	2.41 (2.21, 2.77)	2.03 (1.37, 2.73)	0.19
**Lactosylceramide (Lact-Cer)**				
C14:0 Lact-Cer	10.23 (6.62, 11.37)	9.86 (7.93, 14.01)	10.23 (7.14, 11.37)	0.49
C16:0 Lact-Cer	272.41 (186.65, 319.04)	263.9 (242.73, 275.74)	269.1 (209.73, 314.68)	0.89
C18:0 Lact-Cer	10.55 (8.23, 13.17)	13.71 (11.3, 16.03)	10.56 (8.62, 13.69)	0.21
C18:1 Lact-Cer	6.23 (4.4, 11.23)	6.14 (5.35, 7.09)	6.23 (4.51, 10.54)	0.96
C20:0 Lact-Cer	2.72 (2.1, 3.19)	3.5 (2.27, 4.32)	2.74 (2.1, 3.52)	0.33
C20:1 Lact-Cer	0.24 (0.16, 0.43)	0.33 (0.26, 0.4)	0.25 (0.17, 0.41)	0.38
C22:0 Lact-Cer	8.34 (6.13, 10.46)	9.76 (8.35, 11)	8.66 (6.49, 10.6)	0.43
C22:1 Lact-Cer	0.5 (0.39, 0.74)	0.46 (0.44, 0.51)	0.5 (0.41, 0.7)	0.55
C24:0 Lact-Cer	2.29 (1.83, 2.99)	2.9 (2.19, 3.92)	2.29 (1.86, 3.16)	0.31
C24:1 Lact-Cer	30.34 (24.44, 37.56)	33.25 (25.09, 35.51)	32.07 (24.44, 36.81)	0.93
C26:0 Lact-Cer	0.11 (0.07, 0.14)	0.21 (0.15, 0.24)	0.12 (0.07, 0.14)	***0.018***
C26:1 Lact-Cer	0.11 (0.08, 0.14)	0.1 (0.09, 0.1)	0.11 (0.08, 0.14)	0.52
Total Lact-cer	323.16 (247.73, 420.67)	345.62 (313.98, 361.05)	340.55 (260.45, 413.83)	0.93
**Hexosylceramide (Hex-Cer)**				
C14:0 Hex-Cer	0.5 (0.34, 0.74)	0.7 (0.52, 0.76)	0.52 (0.38, 0.74)	0.4
C16:0 Hex-Cer	75.7 (57.13, 95.44)	71.05 (67.14, 83.92)	72.92 (58.12, 94.92)	0.89
C18:0 Hex-Cer	0.49 (0.39, 0.61)	0.62 (0.41, 0.73)	0.51 (0.39, 0.63)	0.75
C18:1 Hex-Cer	0.19 (0.13, 0.24)	0.17 (0.1, 0.22)	0.19 (0.13, 0.24)	0.52
C20:0 Hex-Cer	0.87 (0.68, 1.12)	0.92 (0.7, 0.92)	0.89 (0.68, 1.06)	0.55
C20:1 Hex-Cer	0.11 (0.07, 0.13)	0.08 (0.04, 0.1)	0.1 (0.06, 0.13)	0.13
C22:0 Hex -Cer	42.26 (34.92, 49.04)	40.79 (26.51, 54.43)	42.26 (34.7, 49.25)	1
C22:1 Hex -Cer	1.46 (1.03, 1.84)	1.44 (1.06, 1.86)	1.46 (1.03, 1.84)	0.93
C24:0 Hex -Cer	56.69 (44.91, 74.04)	64.66 (42.6, 85.72)	56.69 (44.91, 74.99)	0.58
C24:1 Hex -Cer	68.81 (55.5, 88.69)	86.64 (61.15, 110.17)	68.81 (55.5, 90.39)	0.38
C26:0 Hex -Cer	0.88 (0.62, 1.15)	0.97 (0.68, 1.37)	0.88 (0.62, 1.16)	0.52
C26:1 Hex -Cer	0.4 (0.32, 0.56)	0.59 (0.46, 0.68)	0.46 (0.32, 0.61)	0.14
Total Hex-Cer	234.1 (205.53, 296.64)	271.68 (218.23, 324.04)	234.1 (214.22, 301.04)	0.71
**Dihydrosphingosine (dhSph)**	0.53 (0.39, 0.72)	0.4 (0.34, 0.53)	0.52 (0.37, 0.68)	0.23
**Sphingosine**	1.58 (1.33, 2.29)	1.3 (1.17, 1.58)	1.53 (1.27, 2.26)	0.23
**dhSph 1-phosphate (dhSph-1P)**	16.48 (13.28, 19.52)	12.07 (9.92, 15.46)	16.08 (13.12, 19.03)	0.09
**Sphingosine 1-phosphate (S1P)**	56.99 (51.03, 65.53)	47.58 (44.08, 56.37)	56.47 (49.64, 65.39)	0.08
**C16:0 Cer: S1P Ratio**	0.91 (0.64, 1.06)	1.41 (1.02, 1.65)	0.91 (0.65, 1.12)	0.06
**C24:1 Cer: S1P Ratio**	3.97 (3.01, 4.53)	6.2 (5.58, 6.56)	4.07 (3.15, 5.02)	***0.004***
**C16:0 Cer: C24:0 Cer Ratio**	0.09 (0.07, 0.12)	0.1 (0.07, 0.12)	0.09 (0.07, 0.12)	0.96
**C18:0 Cer: C24:0 Cer Ratio**	0.03 (0.02, 0.04)	0.03 (0.02, 0.04)	0.03 (0.02, 0.04)	0.82
**C24:1 Cer: C24:0 Cer Ratio**	0.37 (0.34, 0.46)	0.47 (0.37, 0.52)	0.38 (0.34, 0.5)	0.46

*Females, data presented are median values and interquartile ranges (IQRs), **bold italics**: statistically significant at < 0.05.

## Discussion

In the present study, we examined the concentrations of sphingolipid species in plasma from a well-characterized cohort of predominantly African American female SLE patients as potential biomarkers that can be associated with atherosclerosis severity and/or with clinical SLE disease markers. Defined carotid atherosclerotic plaque burden measurements at baseline and after one year were analyzed for correlations with the baseline concentrations of plasma sphingolipid species. The data demonstrated that TPA and C3 values correlated inversely with concentrations of the two most dominant Lact-Cer species (C16:0 and C24:1) and of Lact-Cer species with long-chain saturated fatty acids. Because lower C3 levels is a marker for increasing SLE disease activity (31), it is then plausible to assume that higher levels of Lact-Cer would be associated with SLE disease activity. As shown in **Table 5**, the correlations between C3 and each of TPA and the change in plaque from baseline are not statistically significant, which is consistent with the characteristics of our study cohort, who is a largely stable SLE population with little activity.

**Table 5:** Correlations between normalized carotid plaque area and markers of dyslipidemia and of SLE disease activity and damage.

	Total plaque area %*	Change in plaque**	Age	Years with SLE	BP Syst.	BP Diast.	Waist-hip ratio	SLEDAI score	SLICC score	LDL-C	HDL-C	TG	C3	C4	dsDNA	Urine protein/creatinine
Total plaque area %*																
Change in plaque**	***-0.61***															
**Age**	***0.44***	-0.01														
**Years with SLE**	***0.34***	-0.01	***0.40***													
**BP Syst.**	-0.07	-0.18	0.29	0.07												
**BP Diast.**	-0.06	0.03	-0.04	-0.09	***0.68***											
**Waist-hip ratio**	-0.11	-0.05	-0.09	0.06	0.14	0.14										
**SLEDAI score**	-0.17	-0.07	-0.27	-0.25	0.21	0.31	-0.08									
**SLICC score**	0.11	***0.36***	***0.33***	***0.36***	0.23	0.20	-0.06	***-0.42***								
**LDL-C**	0.24	0.13	0.14	-0.03	-0.26	-0.02	0.13	-0.001	0.03							
**HDL-C**	0.14	0.21	0.14	-0.03	0.15	0.12	-0.18	0.30	0.12	0.21						
**TG**	0.06	-0.14	0.21	0.05	0.07	0.07	0.08	-0.08	0.002	***0.42***	-0.32					
**C3**	0.26	0.04	***0.37***	0.21	0.05	-0.06	0.27	***-0.35***	***0.39***	0.13	-0.0004	0.06				
**C4**	0.24	0.07	0.02	0.23	-0.02	0.05	***0.33***	***-0.43***	***0.41***	0.09	-0.18	0.20	***0.57***			
**dsDNA**	-0.22	-0.11	-0.16	-0.14	-0.05	0.02	0.01	0.31	***-0.47***	-0.04	-0.14	0.07	***-0.51***	***-0.44***		
**Urine protein/creatinine**	0.21	0.02	0.20	0.17	0.11	0.21	0.04	0.01	0.16	0.17	0.01	0.22	-0.20	-0.05	***0.33***	

**N =39**. Data presented are r values where **bold italics** is statistically significant at < 0.05 **(black)**, < 0.01 **(red)**, < 0.001 **(blue)**, < 0.00001 **(violet)**, & Underlined: p=0.051 to p=0.059.

*% of age- and sex-matched historical controls. **Absolute change in plaque from baseline (visit 1) to visit 2. BP, blood pressure; Syst., systolic; Diast., diastolic; SLEDAI, Systemic lupus erythematosus disease activity index; SLICC, Systemic Lupus International Collaborating Clinics; LDL-; HDL-C, HDL-; LDL-cholesterol; TG, triglycerides.

Lact-Cer is an integral component of the cell outer membrane, serving as a mediator to transduce external stimuli, which may contribute to mortality and morbidity in humans as well as in animal models (recently reviewed in 33). Lact-Cer is synthesized by the action of Lact-Cer synthase, which can be activated by several inflammatory and stress factors. The generated Lact-Cer activates nicotinamide adenine dihydrogen phosphate (NADPH) oxidase producing reactive oxygen species (ROS) and a highly oxidative stress environment, which triggers a cascade of signaling molecules and pathways causing mitochondrial dysfunction and contributing to inflammation, atherosclerosis, CVD, diabetes, and skin conditions (33). Thus, the uncovering of the Lact-Cer-mediated oxidative stress pathway could facilitate our understanding of the progression of atherosclerosis and CVD in SLE. In general, relapsing/remitting SLE disease reduces over time, while long quiescent disease increases. Therefore, the inverse correlation between TPA and the Lact-Cer species observed in this stable SLE cohort may be an indication of prior disease activity, when Lact-Cer may have been elevated. From the current retrospective study, it is not possible to conclude whether increases in Lact-Cer levels would be pathologic. Baseline TPA reflects progression prior to the baseline and may be an accumulation from prior disease flares (with low C3) that have resolved (with normalization of C3). This is consistent with the observations in the Johns Hopkins cohort that combined chronic activity associates with cumulative risk (31). The baseline Lact-Cer species might reflect risk for future progression in the following year in this group of patients with low disease activity.

Sphingolipids are both structural lipids and signaling molecules, and their synthesis and degradation are tightly regulated (8, 9). Cellular accumulations of ceramide have been associated with apoptosis and cell death (8, 34), whereas S1P was found to promote endothelial integrity and lymphocyte migration (35). Sphingolipids in blood are carried on circulating lipoprotein particles (HDL, LDL, and VLDL) and their use as disease biomarkers has been explored (9). Emerging clinical data during the past decade have shown that sphingolipids are of not only ample biochemical interest but also have a possible diagnostic value. Sphingolipids were recently evaluated *via* targeted lipidomics to determine if sphingolipid levels in the circulation would be a valuable cholesterol-independent biomarker for coronary artery disease (CAD) (36). Poss et al. developed a sphingolipids inclusive CAD (SIC) risk score, which was found to have better discriminatory power for CVD than the long-established LDL-C levels (C-statistics of 0.79 and 0.69, respectively) (36). The significance of such a score suggests that plasma sphingolipids could be an added clinical characteristic used to assess more accurately not only the risk, but also the diagnosis and prognosis of CVD in SLE. Future longitudinal studies would evaluate progression of atherosclerotic plaques in patients with SLE earlier in the course of their disease and thus with more disease activity.

In our study, LDL-C concentrations were found to strongly and positively correlate with concentrations of the majority of SM, ceramide, and Hex-Cer species, as well as dihydroceramide, sphingosine, dhSph, C16:0 Cer/S1P and C24:1 Cer/S1P, but we found no significant correlations between concentrations of LDL-C and Lact-Cer species, or between concentrations of HDL-C and any of the plasma sphingolipids. Triglyceride concentrations were found to correlate positively with concentrations of a number of SM and ceramide species, also with dh-Sph and sphingosine, but negatively with only C24:1 Lact-Cer, the second dominant Lact-Cer species. The functional significance of these observations and the possible metabolic pathways behind them are yet to be determined.

Jiang et al. reported lower mean plasma SM levels in whites compared with other ethnic groups (37), and showed that plasma SM is associated with subclinical atherosclerotic disease (38). We have recently reported that healthy African Americans have higher SM levels and lower Lact-Cer levels compared to healthy whites; and that SLE patients, irrespective of race, have higher levels of ceramides, and sphingoid bases and their phosphates compared to healthy participants (19). We also showed that African American SLE patients have higher levels of ceramides, Hex-Cer, sphingosine, and dhSph-1P compared to healthy African Americans. Furthermore, within African American SLE patients, those with atherosclerosis were found to have higher levels of SM (most SM species and total SM) and sphingoid bases (sphingosine and dhSph), lower levels of C24:1 Lact-Cer, and no significant differences in ceramide species levels compared to those without (19). These reports are in accordance with our current data demonstrating that TPA in African American SLE patients correlates positively with C20:0, C22:0 and total SM concentrations, while correlates negatively with concentrations of C24:1 Lact-Cer, several other Lact-Cer species and total Lact-Cer. Remarkably, in the current study, the concentrations of C24:0, C26:0 and C26:1 ceramide species, but not total ceramide correlated positively with TPA. This observation highlights the significance of the determination of individual sphingolipid species levels as a more sensitive-to-change parameter than total levels. Importantly, the longitudinal measurement of TPA shows that TPA increased in size with increased levels of C16:0, C24:1 and total Lact-Cer, and with decreased levels of sphingoid bases (sphingosine and dhSph) and dhSph-1P levels.

A role of the glycosphingolipid pathway in atherosclerosis was previously investigated. Measured concentrations of glycosphingolipids in human aortic intima and media from patients who died of atherosclerosis showed that the level of LacCer was elevated 5-fold compared to unaffected intima (39). Fatty streaks also were found to accumulate several fold higher levels of glycosphingolipids (glucosylceramides, Lact-Cer and GM3) than normal regions of human aorta (40). Concentrations of Lact-Cer species measured in serum from CAD patients were found to be significantly associated with the fatal outcome of CAD, independently of traditional risk factors (32). Furthermore, it was shown that concentration of C18:0 Lact-Cer in plasma from 581 patients, who underwent coronary angiography for acute coronary syndrome (ACS) or stable CAD was associated with vulnerable plaques, as characterized using intra vascular ultra sound virtual histology (IVUS-VH) and near-infrared spectroscopy (NIRS) imaging, and with 1-year major adverse cardiac events (composite endpoint of death or ACS) (41). Since our previous study showed that healthy African Americans have lower plasma Lact-Cer levels compared to healthy whites (19), and our current data show that plasma Lact-Cer levels in African Americans with SLE correlate negatively with TPA, this raises the question whether African Americans have inherent tendency towards accumulating Lact-Cer in tissues or not effluxing Lact-Cer into the circulation. This hypothesis warrants further investigation.

Serum ceramide concentrations have been shown to have independent predictive value for CVD, including CAD, stroke, heart failure and atrial fibrillation (reviewed in 42), despite the fact that a direct cause-effect relationship between CVD and serum ceramide has not been established yet. A ceramide risk score (CERT1) which was based on C16:0, C18:0, and C24:1 ceramide concentrations and their ratios to C24:0 ceramide was developed for clinical use and was found to identify high-risk coronary heart disease patients beyond LDL-C concentration (43, 44). Based on CERT1, patients are stratified into four risk categories, where a linear CVD risk increase is associated with the increasing score both in patients with a stable coronary heart disease and ACS. The CERT1 ceramide score has been implemented in clinical use both in Finland and at Mayo Clinic in the USA (42). In our study, among the three CERT1 ceramide ratios, only C16:0 ceramide/C24:0 ceramide ratio was negatively correlated with TPA (% of control) but positively correlated with change in TPA (*p*< 0.01) **(** **Table 3** **)**. It is important to mention here that TPA% of “disease” controls would be different when compared to a control healthy population.

As an upgrade of CERT1, CERT2 has been recently developed by incorporating distinct phosphatidylcholines (PCs) into the score (45); PCs have been shown to have prognostic value for CVD events (46). The CERT2 score have one ceramide/ceramide ratio, two ceramide/PC ratios and a single PC, whereas the original CERT1 score contains three single ceramides and three ceramide/ceramide ratios. The ceramide-PC ratio components of the CERT2 test showed higher hazard ratios for CVD events than any other ceramide-ceramide ratio (45). The CERT2 score was also significantly associated with inflammatory markers (hs-CRP and IL-6) (47), which suggests that CERT2 could assess both plaque burden and inflammatory residual risk in patients with CVD. Interestingly, patients with renal dysfunction were found to have higher CERT2 scores, while associations of CERT2 with high blood pressure and diabetes were found to be much weaker (47). Furthermore, CERT2 was significantly associated with LDL-C and triglycerides levels; however, CERT2 was prognostic even after adjustment for LDL-C and triglyceride levels (47). In our study, we investigated whether there was any association between circulating creatinine and sphingolipid levels in patients who may have had renal involvement, although creatinine > 3.0 was an exclusion criterion. We found that C22:1, C24:0, C24:1, C26:1 and total Hex-Cers as well as C24:0 Lact-Cer were negatively correlated with serum creatinine (*p*<0.05), but C24:0 SM was positively correlated with serum creatinine (*p*<0.05). Our current data suggest that the use of plasma concentrations of glycosphingolipids (Lact-Cer and Hex-Cer species) with the CERT2 risk estimation tool may improve the stratification of SLE patients for their risk of CVD events.

Fiedorowicz et al. assessed ceramide and S1P serum concentrations in patients with acute ischaemic stroke, transient ischemic attack, and age-matched neurological patients without cerebral ischaemia and recognized the two ratios, S1P/C 24:1 ceramide, and C 24:0 ceramide/C 24:1 ceramide, with a diagnostic potential in ischaemic stroke (48). In our current study, TPA values correlated positively with concentrations of very long-chain ceramide species (C24:0, C26:0 and C26:1). However, C16:0 ceramide/S1P ratio and C24:1 ceramide/S1P ratio correlated positively with LDL-C concentration and the urine protein/creatinine ratio **(** **Table 3** **)**. We have previously reported that SLEDAI significantly correlates with plasma C16:0 ceramide/S1P ratio and C24:1 ceramide/S1P ratio in the African American, but not white SLE patients (19). In this and our previous studies the nephritis comorbidity has been excluded; however, it is possible that the plasma S1P fraction bound to albumin (25) is excreted with the urine (albuminuria), which may alter/inflate the ceramide/S1P ratio possibly causing alterations in correlations with TPA and other clinical variables.

Although this study was not purposely designed to study the effect of statins on the development of atherosclerosis in African American SLE patients, the data showed that ‘undesirably’ C24:1 ceramide and C24:1 ceramide/S1P ratio were significantly higher in the few patients who used statins. The data for statin use in atherosclerosis prevention in SLE, whether in SLE patients or SLE mouse models, has been inconsistent despite reductions in cholesterol levels (reviewed in 6). However, it is reasonable to use statins in SLE patients with traditional CVD risk factors. Therefore, in future studies, statins use could be analyzed as a confounding factor in determining the associations of plasma sphingolipid concentrations with traditional SLE markers. Furthermore, as statins remain the current recommendation to the risk-based approach to CVD treatment, the use of statins in African American SLE patients is not to be discouraged without clinical outcome data; however, the effectiveness of statin treatment to prevent atherosclerosis progression in African American SLE patients warrants further attention. Again, a limitation of our study is that there are only four patients, who were on statins, which makes it difficult to draw any definitive conclusions on the effect of statins on the development of atherosclerosis in African American SLE patients.

In this study, the older the patient the higher the concentrations of long- and very long-chain SM species, the higher the concentrations of C20:0 ceramide species, and the lower the concentrations of long- and very long-chain Lact-Cer species **(** **Table 3** **)**. The duration of SLE prior to visit 1, SLEDAI scores, and concentrations of C4 dsDNA antibodies do not seem to influence the concentrations of plasma sphingolipids. However, C3 concentrations, which were found to correlate negatively with almost all Lact-Cer species, and positively with three long-chain SM species, indicate that these sphingolipid measurements could have an added value in assessing the prognosis of CVD in SLE patients. From the current retrospective study, though it is not possible to infer whether increased Lact-Cer levels would be compensatory or pathologic.

Taken together, the data demonstrate that sphingolipidomics have the potential to be used as an early diagnostic tool of atherosclerosis in SLE and may have an added benefit to the currently available tools in the diagnosis, prognosis, and treatment of the disease. Longitudinal studies are warranted to proof the potential sphingolipid markers for early diagnosis of SLE comorbidities, including CVD.

## Data Availability Statement

The raw data supporting the conclusions of this article will be made available by the authors, without undue reservation.

## Ethics Statement

The studies involving human participants were reviewed and approved by Medical University of South Carolina Institutional Review Board (IRB), (Protocol number: HR1623). The patients/participants provided their written informed consent to participate in this study.

## Author Contributions

SH and JO designed the study. SH administered and supervised the project and wrote the original draft. JO provided the patient plasma samples and the corresponding clinical data. SH and JO validated and interpreted the data. WT and OH contributed to the laboratory work. PN and DW did the statistical analyses. All authors contributed to the article and approved the submitted version.

## Funding

This study was funded by College of Graduate Studies (CGS) and Department of Regenerative Medicine and Cell Biology at MUSC (SH), from South Carolina Clinical & Translational Research (SCTR) (JO), and support from the NIH training grant Research Education Program for Minority Medical Students (REPMMS R25 HL096316) (OH). Sphingolipidomics analyses supported in part by the Lipidomics Shared Resource, Hollings Cancer Center, MUSC (P30 CA138313). This study was also supported by NIH/NCRR MUSC-SCTR Grant number UL1 RR029882. Statistical analysis (PN & DW) supported by three grants from the NIH (NCATS grant # 1UL1TR001450, NIAMS grant # P30-AR072582, and P60 AR062755). The contents are solely the responsibility of the authors and do not necessarily represent the official view of the NIH. Grant funding for this project also came from an award from the VA Research Enhancement Award Program and a grant from the Lupus Foundation. This material is the result of work supported with resources for JO’s time and the use of facilities at the Ralph H. Johnson VA Medical Center. Contents do not represent the views of Department of VA or US Government. The funders had no role in the writing of the manuscript, or in the decision to publish.

## Conflict of Interest

The authors declare that the research was conducted in the absence of any commercial or financial relationships that could be construed as a potential conflict of interest.

## Abbreviations

ACS, Acute coronary syndrome; ACR, American College of Rheumatology; ACE, Angiotensin converting enzyme; ARBs, Angiotensin receptor blockers; dsDNA, Anti-double-stranded DNA; CVD, Cardiovascular Disease; Cer, Ceramide; CERT, Ceramide risk score; CVA, Cerebrovascular accident; CLIA, Clinical Laboratory Improvement Amendments; C3, Complement protein C3; C4, Complement protein C4; CAD, Coronary artery disease; dhSph, Dihydrosphingosine; dhSph-1P, Dihydrosphingosine 1-phosphate; Hex-Cer, Hexosylceramide; HDL-C, High-density lipoprotein cholesterol; IQR, Interquartile range; Lact-Cer, Lactosylceramide; LDL-C, Low-density lipoprotein cholesterol; MUSC, Medical University of South Carolina; MI, Myocardial infarction; PC, Phosphatidylcholine; SIC, Sphingolipids inclusive CAD; SM, Sphingomyelin; S1P, Sphingosine 1-phosphate; SK, Sphingosine kinase; SLE, Systemic lupus erythematosus; SLEDAI, Systemic Lupus Erythematosus Disease Activity Index; SLICC, Systemic Lupus International Collaborating Clinics Damage Index; TPA, Total plaque area; VLDL, Very low-density lipoprotein.

## References

[1] Epidemiology and pathogenesis of systemic lupus erythematosus (2020). Available at: https://www.uptodate.com/contents/epidemiology-and-pathogenesis-of-systemic-lupus-erythematosus?search=systemic%20lupus%20erythematosus&topicRef=4668&source=see_link#H4.

[2] Centers for Disease Control and Prevention (CDC). Systemic Lupus Erythematosus (SLE) (2018). Available at: https://www.cdc.gov/lupus/facts/detailed.html.

[3] The Lupus Foundation of America. Lupus facts and statistics (2017). Available at: https://resources.lupus.org/entry/facts-and-statistics.

[4] Ballou SP Khan MA Kushner I. Clinical Features of Systemic Lupus Erythematosus: Differences Related to Race and Age of Onset. Arthritis Rheum (1982) 25(1):55–60. 10.1002/art.1780250109 6978135

[5] Sherer Y Shoenfeld Y. Mechanisms of Disease: Atherosclerosis in Autoimmune Diseases. Nat Clin Pract Rheumatol (2006) 2:99–106. 10.1038/ncprheum0092 16932663

[6] Skaggs BJ Hahn BH McMahon M. Accelerated Atherosclerosis in Patients With SLE-Mechanisms and Management. Nat Rev Rheumatol (2012) 8:214–23. 10.1038/nrrheum.2012.14 PMC3765069 22331061

[7] Bentley AR Rotimi CN. Interethnic Differences in Serum Lipids and Implications for Cardiometabolic Disease Risk in African Ancestry Populations. Glob Heart (2017) 12:141–50. 10.1016/j.gheart.2017.01.011 PMC5582986 28528248

[8] Hannun YA Obeid LM. Principles of Bioactive Lipid Signalling: Lessons From Sphingolipids. Nat Rev Mol Cell Biol (2008) 9:139–50. 10.1038/nrm2329 18216770

[9] Hammad SM. Blood Sphingolipids in Homeostasis and Pathobiology. Adv Exp Med Biol (2011) 721:57–66. 10.1007/978-1-4614-0650-1_4 21910082

[10] Proia RL Hla T. Emerging Biology of Sphingosine-1-Phosphate: Its Role in Pathogenesis and Therapy. J Clin Invest (2015) 125:1379–87. 10.1172/JCI76369 PMC4409021 25831442

[11] Maceyka M Spiegel S. Sphingolipid Metabolites in Inflammatory Disease. Nature (2014) 510:58–67. 10.1038/nature13475 24899305 PMC4320971

[12] Al Gadban MM German J Truman JP Soodavar F Riemer EC Twal WO. Lack of Nitric Oxide Synthases Increases Lipoprotein Immune Complex Deposition in the Aorta and Elevates Plasma Sphingolipid Levels in Lupus. Cell Immunol (2012) 276:42–51. 10.1016/j.cellimm.2012.03.007 22560558 PMC3399025

[13] McDonald G Deepak S Miguel L Hall CJ Isenberg DA Magee AI. Normalizing Glycosphingolipids Restores Function in CD4+ T Cells From Lupus Patients. J Clin Invest (2014) 124:712–24. 10.1172/JCI69571 PMC3904606 24463447

[14] Nowling TK Mather AR Thiyagarajan T Hernandez-Corbacho MJ Powers TW Jones EE. Renal Glycosphingolipid Metabolism Is Dysfunctional in Lupus Nephritis. J Am Soc Nephrol (2015) 26:1402–13. 10.1681/ASN.2014050508 PMC4446878 25270066

[15] Al Gadban MM Alwan MM Smith KJ Hammad SM. Accelerated Vascular Disease in Systemic Lupus Erythematosus: Role of Macrophage. Clin Immunol (2015) 157:133–44. 10.1016/j.clim.2015.01.008 PMC4410070 25638414

[16] Liu F Li X Yue H Ji J You M Ding L. TLR-Induced SMPD3 Defects Enhance Inflammatory Response of B Cell and Macrophage in the Pathogenesis of SLE. Scand J Immunol (2017) 86:377–88. 10.1111/sji.12611 28889482

[17] Checa A Idborg H Zandian A Sar DG Surowiec I Trygg J. Dysregulations in Circulating Sphingolipids Associate With Disease Activity Indices in Female Patients With Systemic Lupus Erythematosus: A Cross-Sectional Study. Lupus (2017) 26:1023–33. 10.1177/0961203316686707 28134039

[18] Patyna S Buttner S Eckes T Obermuller N Bartel C Braner A. Blood Ceramides as Novel Markers for Renal Impairment in Systemic Lupus Erythematosus. Prostaglandins Other Lipid Mediat (2019) 144:106348. 10.1016/j.prostaglandins.2019.106348 31301404

[19] Hammad SM Hardin JR Wilson DA Twal WO Nietert PJ Oates JC. Race Disparity in Blood Sphingolipidomics Associated With Lupus Cardiovascular Comorbidity. PloS One (2019) 14(11):e0224496. 10.1371/journal.pone.0224496 31747417 PMC6867606

[20] Ravenell RL Kamen DL Spence JD Hollis BW Fleury TJ Janech MG. Premature Atherosclerosis Is Associated With Hypovitaminosis D and Angiotensin-Converting Enzyme Inhibitor Non-Use in Lupus Patients. Am J Med Sci (2012) 344(4):268–73. 10.1097/MAJ.0b013e31823fa7d9 PMC3323721 22222338

[21] Oates JC Ramakrishnan V Nietert PJ Spence JD Fleury TW Markiewicz M. Associations Between Accelerated Atherosclerosis, Oxidized LDL Immune Complexes, and *In Vitro* Endothelial Dysfunction in Systemic Lupus Erythematosus. Trans Am Clin Climatol Assoc (2020) 131:157–77. PMC7358516 32675856

[22] Hochberg MC. Updating the American College of Rheumatology Revised Criteria for the Classification of Systemic Lupus Erythematosus. Arthritis Rheum (1997) 40(9):1725. 10.1002/art.1780400928 9324032

[23] Spence JD Eliasziw M DiCicco M Hackam DG Galil R Lohmann T. Carotid Plaque Area: A Tool for Targeting and Evaluating Vascular Preventive Therapy. Stroke (2002) 33(12):2916–22. 10.1161/01.str.0000042207.16156.b9 12468791

[24] Hammad SM Pierce JS Soodavar F Smith KJ Al Gadban MM Rembiesa B. Blood Sphingolipidomics in Healthy Humans: Impact of Sample Collection Methodology. J Lipid Res (2010) 51:3074–87. 10.1194/jlr.D008532 PMC2936747 20660127

[25] Hammad SM Al Gadban MM Semler AJ Klein RL. Sphingosine 1-Phosphate Distribution in Human Plasma: Associations With Lipid Profiles. J Lipids (2012) 2012:180705. 10.1155/2012/180705 23209911 PMC3503336

[26] Hammad SM Truman JP Al Gadban MM Smith KJ Twal WO Hamner MB. Altered Blood Sphingolipidomics and Elevated Plasma Inflammatory Cytokines in Combat Veterans With Post-Traumatic Stress Disorder. Neurobiol Lipids (2012) 10:2. 24403911 PMC3882130

[27] Klein RL Hammad SM Baker NL Hunt KJ Al Gadban MM Cleary PA. Decreased Plasma Levels of Select Very Long Chain Ceramide Species Are Associated With the Development of Nephropathy in Type 1 Diabetes. Metabolism (2014) 63:1287–95. 10.1016/j.metabol.2014.07.001 PMC5894336 25088746

[28] Lopes-Virella MF Baker NL Hunt KJ Hammad SM Arthur J Virella G. Glycosylated Sphingolipids and Progression to Kidney Dysfunction in Type 1 Diabetes. J Clin Lipidol (2019) 13:481–91. 10.1016/j.jacl.2019.03.00 PMC7218687 31043336

[29] Buie JNJ Hammad SM Nietert PJ Magwood G Adams RJ Bonilha L. Differences in Plasma Levels of Long Chain and Very Long Chain Ceramides Between African Americans and Whites: An Observational Study. PloS One (2019) 14(5):e0216213. 10.1371/journal.pone.0216213 31067249 PMC6505935

[30] Tao CY Shang J Chen T Yu D Jiang YM Liu D. Impact of Antimalarial (AM) on Serum Lipids in Systemic Lupus Erythematosus (SLE) Patients: A Systematic Review and Meta-Analysis. Med (Baltimore) (2019) 98(14):e15030. 10.1097/MD.0000000000015030 PMC6456110 30946340

[31] Petri MA Barr E Magder LS. Development of a Systemic Lupus Erythematosus Cardiovascular Risk Equation. Lupus Sci Med (2019) 6(1):e000346. 10.1136/lupus-2019-000346 31749976 PMC6827738

[32] Tarasov K Ekroos K Suoniemi M Kauhanen D Sylvänne T Hurme R. Molecular Lipids Identify Cardiovascular Risk and Are Efficiently Lowered by Simvastatin and PCSK9 Deficiency. J Clin Endocrinol Metab (2014) 99(1):E45–52. 10.1210/jc.2013-2559 PMC3928964 24243630

[33] Chatterjee S Balram A Li W. Convergence: Lactosylceramide-Centric Signaling Pathways Induce Inflammation, Oxidative Stress, and Other Phenotypic Outcomes. Int J Mol Sci (2021) 22:1816. 10.3390/ijms22041816 33673027 PMC7917694

[34] Hannun YA Obeid LM. Many Ceramides. J Biol Chem (2011) 286:27855–62. 10.1074/jbc.R111.254359 PMC3151029 21693702

[35] Matloubian M Lo CG Cinamon G Lesneski MJ Xu Y Brinkmann V. Lymphocyte Egress From Thymus and Peripheral Lymphoid Organs Is Dependent on S1P Receptor 1. Nature (2004) 427:355–60. 10.1038/nature02284 14737169

[36] Poss AM Maschek JA Cox JE Hauner BJ Hopkins PN Hunt SC. Machine Learning Reveals Serum Sphingolipids as Cholesterol-Independent Biomarkers of Coronary Artery Disease. J Clin Invest (2020) 130:1363–76. 10.1172/JCI131838 PMC7269567 31743112

[37] Jiang XC Paultre F Pearson TA Reed RG Francis CK Lin M. Plasma Sphingomyelin Level as a Risk Factor for Coronary Artery Disease. Arterioscler Thromb Vasc Biol (2000) 20:2614–18. 10.1161/01.atv.20.12.2614 11116061

[38] Nelson JC Jiang XC Tabas I Tall A Shea S. Plasma Sphingomyelin and Subclinical Atherosclerosis: Findings From the Multi-Ethnic Study of Atherosclerosis. Am J Epidemiol (2006) 163:903–12. 10.1093/aje/kwj140 16611667

[39] Chatterjee SB Dey S Shi WY Thomas K Hutchins GM. Accumulation of Glycosphingolipids in Human Atherosclerotic Plaque and Unaffected Aorta Tissues. Glycobiology (1997) 7(1):57–65. 10.1093/glycob/7.1.57 9061365

[40] Mukhin DN Chao FF Kruth HS. Glycosphingolipid Accumulation in the Aortic Wall Is Another Feature of Human Atherosclerosis. Arterioscler Thromb Vasc Biol (1995) 15(10):1607–15. 10.1161/01.atv.15.10.1607 7583534

[41] Cheng JM Suoniemi M Kardys I Vihervaara T de Boer SP Akkerhuis KM. Plasma Concentrations of Molecular Lipid Species in Relation to Coronary Plaque Characteristics and Cardiovascular Outcome: Results of the ATHEROREMO-IVUS Study. Atherosclerosis (201) 243(2):560–66. 10.1016/j.atherosclerosis.2015.10.022 26523994

[42] Hilvo M Vasile VC Donato LJ Hurme R Laaksonen R. Ceramides and Ceramide Scores: Clinical Applications for Cardiometabolic Risk Stratification. Front Endocrinol (Lausanne) (2020) 11:570628. 10.3389/fendo.2020.570628 33133018 PMC7550651

[43] Laaksonen R Ekroos K Sysi-Aho M Hilvo M Vihervaara T Kauhanen D. Plasma Ceramides Predict Cardiovascular Death in Patients With Stable Coronary Artery Disease and Acute Coronary Syndromes Beyond LDL Cholesterol. Eur Heart J (2016) 37:1967–76. 10.1093/eurheartj/ehw148 PMC4929378 27125947

[44] Havulinna AS Sysi-Aho M Hilvo M Kauhanen D Hurme R Ekroos K. Circulating Ceramides Predict Cardiovascular Outcomes in the Population Based FINRISK 2002 Cohort. Arterioscler Thromb Vasc Biol (2016) 36:2424–30. 10.1161/ATVBAHA.116.307497 27765765

[45] Hilvo M Meikle PJ Pedersen ER Tell GS Dhar I Brenner H. Development and Validation of a Ceramide- and Phospholipid-Based Cardiovascular Risk Estimation Score for Coronary Artery Disease Patients. Eur Heart J (2020) 41(3):371–80. 10.1093/eurheartj/ehz387 31209498

[46] Mundra PA Barlow CK Nestel PJ Barnes EH Kirby A Thompson P. Large-Scale Plasma Lipidomic Profiling Identifies Lipids That Predict Cardiovascular Events in Secondary Prevention. JCI Insight (2018) 3:e121326. 10.1172/jci.insight.121326 PMC6171797 30185661

[47] Hilvo M Wallentin L Ghukasyan Lakic T Held C Kauhanen D Jylhä A. Prediction of Residual Risk by Ceramide-Phospholipid Score in Patients With Stable Coronary Heart Disease on Optimal Medical Therapy. J Am Heart Assoc (2020) 9(10):e015258. 10.1161/JAHA.119.015258 32375553 PMC7660846

[48] Fiedorowicz A Kozak-Sykała A Bobak Ł Kałas W Strządała L. Ceramides and Sphingosine-1-Phosphate as Potential Markers in Diagnosis of Ischaemic Stroke. Neurol Neurochir Pol (2019) 53(6):484–91. 10.5603/PJNNS.a2019.0063 31804702

