# Supplementary material for: Plasma Sphingolipid Profile Associated With Subclinical Atherosclerosis and Clinical Disease Markers of Systemic Lupus Erythematosus: Potential Predictive Value
Source: Front Immunol. 2021 Jul 21;12:694318. doi: 10.3389/fimmu.2021.694318 (PMC8335560; doi:10.3389/fimmu.2021.694318)
Supplement: Supplementary file 1 [file Table_1.docx]

**TABLE S1: Comparisons in plasma sphingolipids between patients who did versus did not use angiotensin converting enzyme (ACE) inhibitors**

| **Sphingolipids**  **[pmol/100 µl plasma]** | **ACE use not noted**  **N =30** | | **ACE use noted**  **N =9** | | ***P value*** |
| --- | --- | --- | --- | --- | --- |
| **Sphingomyelin (SM)** |  |  |  |  |  |
| C14:0 SM | 1,028.5 | ± 349.9 | 1,159.0 | ± 472.0 | 0.37 |
| C16:0 SM | 18,577.2 | ± 3777.8 | 19,133.3 | ± 4149.7 | 0.71 |
| C18:0 SM | 1,333.4 | ± 227.3 | 1,368.3 | ± 172.2 | 0.67 |
| C18:1 SM | 656.5 | ± 131.4 | 698.1 | ± 133.6 | 0.41 |
| C20:0 SM | 726.4 | ± 121.4 | 694.5 | ± 87.0 | 0.47 |
| C20:1 SM | 295.4 | ± 52.1 | 291.4 | ± 35.3 | 0.83 |
| C22:0 SM | 1,415.8 | ± 264.5 | 1,372.3 | ± 162.9 | 0.64 |
| C22:1 SM | 1,072.8 | ± 145.2 | 1,100.8 | ± 132.4 | 0.61 |
| C24:0 SM | 1,238.8 | ± 263.1 | 1,153.0 | ± 157.6 | 0.36 |
| C24:1 SM | 2,983.9 | ± 352.4 | 2,974.1 | ± 338.2 | 0.94 |
| C26 SM | 8.7 | ± 1.6 | 7.9 | ± 2.7 | 0.43 |
| C26:1 SM | 22.0 | ± 5.0 | 18.5 | ± 4.5 | 0.07 |
| Total SM | 29,359.4 | ± 4593.6 | 29,971.1 | ± 5023.6 | 0.73 |
|  |  |  |  |  |  |
| **Ceramide (Cer)** |  |  |  |  |  |
| C14:0 Cer | 3.1 | ± 0.8 | 3.8 | ± 1.1 | ***0.04*** |
| C16:0 Cer | 50.3 | ± 18.5 | 70.4 | ± 25.2 | ***0.01*** |
| C18:0 Cer | 15.3 | ± 6.6 | 21.0 | ± 7.6 | ***0.04*** |
| C18:1 Cer | 4.8 | ± 2.6 | 6.6 | ± 2.8 | 0.09 |
| C20:0 Cer | 31.8 | ± 10.6 | 41.5 | ± 22.9 | 0.25 |
| C20:1 Cer | 6.0 | ± 2.3 | 7.5 | ± 3.2 | 0.12 |
| C20:4 Cer | 0.04 | ± 0.03 | 0.03 | ± 0.01 | 0.07 |
| C22:0 Cer | 135.1 | ± 35.3 | 156.7 | ± 28.4 | 0.10 |
| C22:1 Cer | 48.7 | ± 12.8 | 60.9 | ± 14.7 | ***0.02*** |
| C24:0 Cer | 578.5 | ± 190.8 | 647.4 | ± 215.2 | 0.36 |
| C24:1 Cer | 223.0 | ± 58.1 | 278.8 | ± 78.5 | ***0.03*** |
| C26:0 Cer | 18.8 | ± 10.2 | 18.4 | ± 7.2 | 0.90 |
| C26:1 Cer | 10.1 | ± 4.6 | 9.7 | ± 3.0 | 0.80 |
| Total Cer | 1,125.6 | ± 314.1 | 1,322.6 | ± 345.8 | 0.12 |
| **Dihydro-C16:0 Cer** | 2.1 | ± 1.0 | 2.4 | ± 1.2 | 0.35 |
|  |  |  |  |  |  |
| **Lactosylceramide**  **(Lact-Cer)** |  |  |  |  |  |
| C14:0 Lact-Cer | 9.9 | ± 4.7 | 9.5 | ± 2.7 | 0.83 |
| C16:0 Lact-Cer | 264.0 | ± 86.6 | 268.2 | ± 92.8 | 0.90 |
| C18:0 Lact-Cer | 11.5 | ± 4.0 | 12.0 | ± 6.0 | 0.76 |
| C18:1 Lact-Cer | 8.0 | ± 4.4 | 7.6 | ± 3.9 | 0.84 |
| C20:0 Lact-Cer | 2.9 | ± 1.3 | 2.7 | ± 1.0 | 0.54 |
| C20:1 Lact-Cer | 0.3 | ± 0.2 | 0.2 | ± 0.2 | 0.27 |
| C22:0 Lact-Cer | 8.8 | ± 3.4 | 9.2 | ± 3.7 | 0.73 |
| C22:1 Lact-Cer | 0.6 | ± 0.3 | 0.5 | ± 0.2 | 0.40 |
| C24:0 Lact-Cer | 2.5 | ± 0.9 | 2.7 | ± 1.0 | 0.48 |
| C24:1 Lact-Cer | 31.8 | ± 11.0 | 31.8 | ± 13.9 | 1.00 |
| C26:0 Lact-Cer | 0.1 | ± 0.1 | 0.1 | ± 0.1 | 0.31 |
| C26:1 Lact-Cer | 0.1 | ± 0.04 | 0.1 | ± 0.04 | 0.27 |
| Total Lact-cer | 340.5 | ± 107.8 | 344.8 | ± 113.6 | 0.92 |
|  |  |  |  |  |  |
| **Hexosylceramide**  **(Hex-Cer)** |  |  |  |  |  |
| C14:0 Hex-Cer | 0.6 | ± 0.3 | 0.6 | ± 0.3 | 0.60 |
| C16:0 Hex-Cer | 75.4 | ± 27.3 | 80.9 | ± 27.5 | 0.60 |
| C18:0 Hex-Cer | 0.6 | ± 0.3 | 0.5 | ± 0.1 | 0.29 |
| C18:1 Hex-Cer | 0.2 | ± 0.1 | 0.2 | ± 0.1 | 0.62 |
| C20:0 Hex-Cer | 1.0 | ± 0.4 | 0.8 | ± 0.2 | 0.32 |
| C20:1 Hex-Cer | 0.1 | ± 0.1 | 0.1 | ± 0.1 | 0.63 |
| C22:0 Hex -Cer | 41.7 | ± 11.5 | 40.5 | ± 12.4 | 0.79 |
| C22:1 Hex -Cer | 1.6 | ± 0.6 | 1.3 | ± 0.4 | 0.20 |
| C24:0 Hex -Cer | 57.7 | ± 16.4 | 59.0 | ± 16.7 | 0.84 |
| C24:1 Hex -Cer | 75.4 | ± 24.8 | 70.5 | ± 21.3 | 0.60 |
| C26:0 Hex -Cer | 0.9 | ± 0.4 | 0.9 | ± 0.4 | 0.91 |
| C26:1 Hex -Cer | 0.5 | ± 0.2 | 0.5 | ± 0.2 | 0.81 |
| Total Hex-Cer | 255.6 | ± 67.4 | 255.7 | ± 61.4 | 0.995 |
|  |  |  |  |  |  |
| **Dihydrosphingosine**  **(dhSph)** | 0.5 | ± 0.2 | 0.7 | ± 0.3 | 0.16 |
| **Sphingosine** | 1.7 | ± 0.7 | 2.1 | ± 0.9 | 0.16 |
| **dhSph 1-phosphate**  **(dhSph-1P)** | 15.9 | ± 4.3 | 15.6 | ± 2.8 | 0.88 |
| **Sphingosine 1-**  **phosphate (S1P)** | 59.4 | ± 13.9 | 58.4 | ± 6.9 | 0.77 |
| **C16:0 Cer : S1P Ratio** | 0.9 | ± 0.4 | 1.2 | ± 0.4 | ***0.04*** |
| **C24:1 Cer : S1P Ratio** | 3.9 | ± 1.3 | 4.9 | ± 1.6 | 0.08 |
| **C16:0 Cer : C24:0 Cer Ratio** | 0.1 | ± 0.03 | 0.1 | ± 0.04 | 0.05 |
| **C18:0 Cer : C24:0 Cer Ratio** | 0.03 | ± 0.01 | 0.03 | ± 0.01 | 0.13 |
| **C24:1 Cer : C24:0 Cer Ratio** | 0.4 | ± 0.1 | 0.4 | ± 0.1 | 0.24 |
| **D**ata presented are mean values and standard deviation, ***bold italics***: statistically significant at < 0.05 | | | | | |
