# Supplementary material for: Plasma Sphingolipid Profile Associated With Subclinical Atherosclerosis and Clinical Disease Markers of Systemic Lupus Erythematosus: Potential Predictive Value
Source: Front Immunol. 2021 Jul 21;12:694318. doi: 10.3389/fimmu.2021.694318 (PMC8335560; doi:10.3389/fimmu.2021.694318)
Supplement: Supplementary file 2 [file Table_2.docx]

**TABLE S2: Comparisons in plasma sphingolipids between patients who did versus did not use angiotensin receptor blockers (ARBs)**

| **Sphingolipids**  **[pmol/100 µl plasma]** | **ARBs use not noted**  **N =36** | | **ARBs use noted**  **N =3** | | ***P value*** |
| --- | --- | --- | --- | --- | --- |
| **Sphingomyelin (SM)** |  |  |  |  |  |
| C14:0 SM | 1,061.9 | ± 389.0 | 1,018.9 | ± 274.6 | 0.85 |
| C16:0 SM | 18,604.0 | ± 3863.4 | 19,924.4 | ± 3642.2 | 0.57 |
| C18:0 SM | 1,343.0 | ± 217.2 | 1,323.9 | ± 214.8 | 0.88 |
| C18:1 SM | 657.9 | ± 123.7 | 763.7 | ± 211.0 | 0.18 |
| C20:0 SM | 729.7 | ± 112.2 | 590.7 | ± 23.0 | ***0.04*** |
| C20:1 SM | 292.4 | ± 48.1 | 320.1 | ± 52.9 | 0.35 |
| C22:0 SM | 1,426.8 | ± 240.5 | 1,153.9 | ± 106.8 | 0.06 |
| C22:1 SM | 1,077.3 | ± 146.3 | 1,102.7 | ± 57.1 | 0.77 |
| C24:0 SM | 1,236.8 | ± 245.3 | 1,004.2 | ± 25.4 | ***<0.0001*** |
| C24:1 SM | 2,994.6 | ± 355.0 | 2,825.6 | ± 119.0 | 0.42 |
| C26 SM | 8.6 | ± 2.0 | 8.0 | ± 0.05 | 0.11 |
| C26:1 SM | 21.5 | ± 5.1 | 17.1 | ± 3.0 | 0.15 |
| Total SM | 29,454.5 | ± 4725.7 | 30,053.3 | ± 4111.8 | 0.83 |
|  |  |  |  |  |  |
| **Ceramide (Cer)** |  |  |  |  |  |
| C14:0 Cer | 3.3 | ± 1.0 | 3.3 | ± 0.2 | 0.97 |
| C16:0 Cer | 53.9 | ± 21.7 | 67.4 | ± 20.0 | 0.30 |
| C18:0 Cer | 16.2 | ± 7.0 | 21.1 | ± 9.9 | 0.27 |
| C18:1 Cer | 5.0 | ± 2.5 | 7.6 | ± 3.7 | 0.11 |
| C20:0 Cer | 34.0 | ± 15.2 | 34.8 | ± 5.3 | 0.93 |
| C20:1 Cer | 6.2 | ± 2.5 | 8.1 | ± 3.4 | 0.24 |
| C20:4 Cer | 0.04 | ± 0.03 | 0.04 | ± 0.03 | 0.82 |
| C22:0 Cer | 141.9 | ± 35.3 | 117.8 | ± 18.9 | 0.25 |
| C22:1 Cer | 51.2 | ± 14.3 | 55.5 | ± 11.3 | 0.61 |
| C24:0 Cer | 605.7 | ± 196.0 | 458.8 | ± 163.9 | 0.22 |
| C24:1 Cer | 235.6 | ± 66.8 | 238.9 | ± 79.1 | 0.94 |
| C26:0 Cer | 19.1 | ± 9.6 | 14.5 | ± 8.8 | 0.43 |
| C26:1 Cer | 10.1 | ± 4.3 | 8.7 | ± 3.9 | 0.59 |
| Total Cer | 1,182.2 | ± 331.5 | 1,036.4 | ± 301.6 | 0.47 |
| **Dihydro-C16:0 Cer** | 2.2 | ± 1.0 | 1.8 | ± 0.4 | 0.59 |
|  |  |  |  |  |  |
| **Lactosylceramide**  **(Lact-Cer)** |  |  |  |  |  |
| C14:0 Lact-Cer | 9.8 | ± 4.4 | 8.9 | ± 1.8 | 0.72 |
| C16:0 Lact-Cer | 265.8 | ± 90.0 | 254.5 | ± 36.2 | 0.83 |
| C18:0 Lact-Cer | 11.6 | ± 4.6 | 11.6 | ± 1.8 | 0.99 |
| C18:1 Lact-Cer | 7.9 | ± 4.4 | 7.2 | ± 2.4 | 0.77 |
| C20:0 Lact-Cer | 2.9 | ± 1.2 | 2.6 | ± 1.0 | 0.67 |
| C20:1 Lact-Cer | 0.3 | ± 0.2 | 0.2 | ± 0.05 | 0.33 |
| C22:0 Lact-Cer | 8.8 | ± 3.5 | 9.6 | ± 2.6 | 0.72 |
| C22:1 Lact-Cer | 0.6 | ± 0.3 | 0.5 | ± 0.1 | 0.68 |
| C24:0 Lact-Cer | 2.5 | ± 0.9 | 2.7 | ± 1.4 | 0.78 |
| C24:1 Lact-Cer | 31.8 | ± 12.0 | 31.7 | ± 1.9 | 0.94 |
| C26:0 Lact-Cer | 0.1 | ± 0.1 | 0.2 | ± 0.1 | 0.35 |
| C26:1 Lact-Cer | 0.1 | ± 0.04 | 0.1 | ± 0.01 | 0.53 |
| Total Lact-cer | 342.5 | ± 111.8 | 329.7 | ± 35.2 | 0.85 |
|  |  |  |  |  |  |
| **Hexosylceramide**  **(Hex-Cer)** |  |  |  |  |  |
| C14:0 Hex-Cer | 0.6 | ± 0.3 | 0.6 | ± 0.1 | 0.98 |
| C16:0 Hex-Cer | 75.8 | ± 27.5 | 87.3 | ± 23.5 | 0.49 |
| C18:0 Hex-Cer | 0.6 | ± 0.2 | 0.5 | ± 0.2 | 0.71 |
| C18:1 Hex-Cer | 0.2 | ± 0.1 | 0.2 | ± 0.02 | 0.59 |
| C20:0 Hex-Cer | 1.0 | ± 0.4 | 0.9 | ± 0.3 | 0.76 |
| C20:1 Hex-Cer | 0.1 | ± 0.1 | 0.1 | ± 0.02 | 0.77 |
| C22:0 Hex -Cer | 41.1 | ± 11.1 | 45.1 | ± 18.5 | 0.57 |
| C22:1 Hex -Cer | 1.5 | ± 0.5 | 1.5 | ± 0.6 | 0.89 |
| C24:0 Hex -Cer | 57.3 | ± 15.6 | 66.1 | ± 25.9 | 0.37 |
| C24:1 Hex -Cer | 73.1 | ± 23.2 | 88.9 | ± 33.4 | 0.27 |
| C26:0 Hex -Cer | 0.9 | ± 0.4 | 0.9 | ± 0.5 | 0.85 |
| C26:1 Hex -Cer | 0.5 | ± 0.2 | 0.5 | ± 0.2 | 0.74 |
| Total Hex-Cer | 252.5 | ± 64.3 | 292.7 | ± 80.8 | 0.31 |
|  |  |  |  |  |  |
| **Dihydrosphingosine**  **(dhSph)** | 0.6 | ± 0.3 | 0.4 | ± 0.1 | 0.34 |
| **Sphingosine** | 1.8 | ± 0.8 | 1.4 | ± 0.2 | 0.27 |
| **dhSph 1-phosphate**  **(dhSph-1P)** | 16.1 | ± 4.0 | 12.6 | ± 2.2 | 0.14 |
| **Sphingosine 1-**  **phosphate (S1P)** | 60.0 | ± 12.6 | 49.3 | ± 7.9 | 0.16 |
| **C16:0 Cer : S1P Ratio** | 0.9 | ± 0.4 | 1.4 | ± 0.3 | 0.07 |
| **C24:1 Cer : S1P Ratio** | 4.1 | ± 1.4 | 4.9 | ± 1.7 | 0.35 |
| **C16:0 Cer : C24:0 Cer Ratio** | 0.1 | ± 0.03 | 0.2 | ± 0.02 | ***0.001*** |
| **C18:0 Cer : C24:0 Cer Ratio** | 0.03 | ± 0.01 | 0.05 | ± 0.01 | ***0.01*** |
| **C24:1 Cer : C24:0 Cer Ratio** | 0.4 | ± 0.1 | 0.5 | ± 0.03 | ***0.02*** |
| **D**ata presented are mean values and standard deviation, ***bold italics***: statistically significant at < 0.05 | | | | | |
